# Supplementary material for: Multi‐dataset identification of innovative feature genes and molecular mechanisms in keratoconus
Source: J Cell Mol Med. 2024 Sep 19;28(18):e70079. doi: 10.1111/jcmm.70079 (PMC11412914; doi:10.1111/jcmm.70079)
Supplement: Supplementary file 2 — Material S2. [file JCMM-28-e70079-s002.zip › 7.GSEA analysis/1.ARL11/KEGG_ETHER_LIPID_METABOLISM.html]

Details for gene set KEGG\_ETHER\_LIPID\_METABOLISM[GSEA]

|  || Dataset | ARL11.group.cls#h\_versus\_l |
| Phenotype | group.cls#h\_versus\_l |
| Upregulated in class | l |
| GeneSet | KEGG\_ETHER\_LIPID\_METABOLISM |
| Enrichment Score (ES) | -0.46982917 |
| Normalized Enrichment Score (NES) | -1.21519 |
| Nominal p-value | 0.21960784 |
| FDR q-value | 1.0 |
| FWER p-Value | 0.982 |
Table: GSEA Results Summary

  

Fig 1: Enrichment plot: KEGG\_ETHER\_LIPID\_METABOLISM      
 Profile of the Running ES Score & Positions of GeneSet Members on the Rank Ordered List

  

| SYMBOL | TITLE | RANK IN GENE LIST | RANK METRIC SCORE | RUNNING ES | CORE ENRICHMENT || 1 | PLA2G7 | na | 931 | 0.397 | 0.0904 | No |
| 2 | PLA2G4E | na | 4751 | 0.174 | 0.0316 | No |
| 3 | PLA2G3 | na | 6310 | 0.129 | 0.0246 | No |
| 4 | PLA2G12B | na | 9215 | 0.072 | -0.0380 | No |
| 5 | PLD2 | na | 12343 | 0.033 | -0.1184 | No |
| 6 | PLA2G2C | na | 13424 | 0.024 | -0.1425 | No |
| 7 | PAFAH1B1 | na | 14081 | 0.018 | -0.1561 | No |
| 8 | JMJD7-PLA2G4B | na | 14509 | 0.015 | -0.1640 | No |
| 9 | PLD1 | na | 14860 | 0.012 | -0.1705 | No |
| 10 | AGPS | na | 15082 | 0.011 | -0.1738 | No |
| 11 | LPCAT2 | na | 15730 | 0.006 | -0.1908 | No |
| 12 | PAFAH1B3 | na | 16280 | 0.002 | -0.2061 | No |
| 13 | PLA2G4A | na | 17245 | -0.005 | -0.2324 | No |
| 14 | PLA2G6 | na | 17692 | -0.008 | -0.2428 | No |
| 15 | PLA2G12A | na | 17822 | -0.009 | -0.2439 | No |
| 16 | PLA2G2E | na | 19951 | -0.023 | -0.2983 | No |
| 17 | PAFAH2 | na | 20444 | -0.027 | -0.3045 | No |
| 18 | LPCAT4 | na | 20674 | -0.029 | -0.3026 | No |
| 19 | PLA2G10 | na | 23788 | -0.060 | -0.3747 | No |
| 20 | ENPP6 | na | 24767 | -0.073 | -0.3813 | No |
| 21 | PAFAH1B2 | na | 27839 | -0.127 | -0.4324 | Yes |
| 22 | PLA2G2D | na | 27985 | -0.130 | -0.3981 | Yes |
| 23 | CHPT1 | na | 29637 | -0.177 | -0.3936 | Yes |
| 24 | PLA2G2F | na | 30623 | -0.208 | -0.3605 | Yes |
| 25 | PLA2G5 | na | 32093 | -0.264 | -0.3250 | Yes |
| 26 | PLA2G2A | na | 32310 | -0.275 | -0.2502 | Yes |
| 27 | ENPP2 | na | 33125 | -0.331 | -0.1760 | Yes |
| 28 | PLA2G1B | na | 33526 | -0.362 | -0.0807 | Yes |
| 29 | LPCAT1 | na | 33814 | -0.391 | 0.0263 | Yes |
Table: GSEA details [plain text format]

  

Fig 2: KEGG\_ETHER\_LIPID\_METABOLISM      
 Blue-Pink O' Gram in the Space of the Analyzed GeneSet

  

Fig 3: KEGG\_ETHER\_LIPID\_METABOLISM: Random ES distribution      
 Gene set null distribution of ES for **KEGG\_ETHER\_LIPID\_METABOLISM**
